# Structure–Property Relationships in Streptomycin Sulfate–Incorporated Bioactive Glass/Chitosan Composite Scaffold: Physicochemical and Antibacterial Insights

**DOI:** 10.3390/polym18101251

**Published:** 2026-05-21

**Authors:** Abdelrahman G. Gadallah, Ahmed A. Bhran, M. A. Farag, A. S. Abdraboh, A. A. Al-Esnawy

**Affiliations:** 1 Chemical Engineering Department, College of Engineering, Imam Mohammad Ibn Saud Islamic University (IMSIU), Riyadh 11432, Saudi Arabia; agadallah@imamu.edu.sa; 2 Physics Department, Faculty of Science, Al-Azhar University, Cairo 11884, Egypt; moha_far2010@yahoo.com (M.A.F.); ah_biophysics6@azhar.edu.eg (A.S.A.); a.alesnawy@azhar.edu.eg (A.A.A.-E.)

**Keywords:** bioactive glass, chitosan composites, streptomycin sulfate, structure–property relationships, FTIR spectroscopy, X-ray diffraction, freeze-drying

## Abstract

In this study, a streptomycin sulfate-loaded bioactive glass/chitosan (STRS–BG/CH) composite scaffold was fabricated via an improved unidirectional freeze-drying method, with drug loadings of 20–40%. The scaffolds were investigated by X-ray diffraction, Fourier transform infrared spectroscopy, scanning electron microscopy, and energy dispersive X-ray analysis before and after in vitro testing. Antibacterial efficacy was evaluated against Gram-positive (*Enterococcus faecalis*, *Staphylococcus aureus*) and Gram-negative (*Klebsiella pneumoniae*, *Escherichia coli*) microorganisms via the agar diffusion method. The STRS–BG/CH scaffolds exhibited highly interconnected porous structures, prolonged antibacterial activity, and enhanced apatite-forming ability in vitro. Compared with bead-based carriers, scaffold-based systems provide enhanced structural integrity and interconnected porosity, which are advantageous for sustained drug release, apatite formation, and tissue integration. Accordingly, these multifunctional scaffolds may simultaneously provide localized antibacterial activity and potential relevance to bone tissue engineering applications. The prepared STRS–BG/CH scaffolds functioned as controlled release carriers for streptomycin sulfate while simultaneously maintaining antibacterial efficacy and bioactive performance. These results illustrate the importance of STRS–BG/CH scaffolds as a promising antibacterial bioactive scaffold system, warranting further biological investigation.

## 1. Introduction

Streptomycin sulfate (STRS) is a broad-spectrum aminoglycoside antibiotic exhibiting potent antibacterial activity against both Gram-positive and Gram-negative microorganisms. Although streptomycin is clinically effective for the treatment of bacterial infections, systemic administration may lead to adverse effects such as nephrotoxicity and ototoxicity while limiting the maintenance of therapeutic drug concentrations at localized defect sites [1,2,3]. Previous studies have reported that STRS-loaded 58S-BG/CH composite beads can function as localized drug delivery systems with notable antibacterial activity. However, the relatively rapid release of the antibiotic from bead-based systems may limit their ability to provide sustained antibacterial effects [4,5].

Bead-based systems are effective carriers for pharmaceutical loading; however, their discrete particulate nature limits the formation of mechanically stable three-dimensional architectures required for enhanced hydroxyapatite formation, cellular infiltration, and long-term tissue integration. Due to these design restrictions, bead-based systems are not as effective for applications that require directed bone regeneration [6,7].

In contrast, scaffold-based systems possess a highly interconnected porous structure that facilitates ion transport, promotes hydroxyapatite formation, and enhances interactions with surrounding biological tissues. These features are essential for improving bioactivity and osteoconductivity. Compared to bead-based carriers, scaffold systems typically exhibit extended drug release durations ranging from several days to weeks, whereas bead-based systems often release their payload within hours to a few days. While this sustained release profile supports prolonged antibacterial activity, optimizing the balance between antibacterial efficacy and bioactivity remains a key challenge.

Therefore, the development of scaffold-based systems with controlled drug release and enhanced multifunctional performance remains an important objective in bone tissue engineering. Compared with dense matrices and particulate delivery systems, scaffold-based biomaterials provide interconnected porous architectures that facilitate ion transport, sustained localized drug release, and apatite formation. These characteristics make scaffold systems attractive candidates for multifunctional bone tissue engineering applications [8,9].

Freeze-drying (lyophilization) is a widely used technique for the fabrication of highly porous scaffolds. The sublimation of frozen solvents during lyophilization enables the formation of interconnected three-dimensional architectures suitable for tissue engineering and controlled drug delivery applications. Such porous structures facilitate drug incorporation, sustained release behavior, nutrient transport, and tissue ingrowth while preserving the structural integrity required for regenerative applications [10].

Chitosan (CH) is among the most extensively investigated natural polymers for scaffold fabrication due to its biocompatibility, biodegradability, intrinsic antibacterial activity, and excellent drug-loading capability [11]. The presence of protonated amino groups within the chitosan structure facilitates electrostatic interactions with ionic therapeutic agents, thereby enabling efficient drug incorporation and controlled release behavior. Moreover, freeze-dried chitosan-based scaffolds typically exhibit highly interconnected porous networks that support drug diffusion, nutrient transport, and tissue ingrowth [12].

This study aims to systematically investigate the structure–property–function relationships governing scaffold performance. Streptomycin sulfate-loaded 60S bioactive glass/chitosan (STRS-60S-BG/CH) composite scaffolds were fabricated using the freeze-drying technique to evaluate the influence of STRS loading on scaffold morphology, antibacterial performance, bioactivity, and drug release behavior. Particular emphasis was placed on correlating scaffold composition and microstructure with sustained antibacterial efficacy and in vitro apatite-forming ability. In addition, the developed scaffold-based systems were compared with previously reported bead-based delivery systems to highlight their advantages in controlled release and structural stability for bone tissue engineering applications. Compared with dense matrices and particulate delivery systems, scaffold-based biomaterials provide interconnected porous architectures that facilitate ion transport, sustained localized drug release, and apatite formation. These characteristics make scaffold systems attractive candidates for multifunctional biomaterial applications.

## 2. Materials and Methods

### 2.1. Materials

Tetraethyl orthosilicate (TEOS, C_8_H_20_O_4_Si, Mw = 208.33 g/mol, ≥99% purity, Merck, Darmstadt, Germany), calcium nitrate tetrahydrate (Ca(NO_3_)_2_·4H_2_O, Mw = 236.15 g/mol, ≥99% purity, Merck, Darmstadt, Germany), and triethyl phosphate (TEP, C_6_H_15_O_4_P, Mw = 182.15 g/mol, ≥99% purity, Merck, Darmstadt, Germany) were used as precursors for bioactive glass synthesis. Nitric acid (HNO_3_, 2 M aqueous solution, Merck, Darmstadt, Germany) was used as a catalyst. Chitosan (CH, C_56_H_103_N_9_O_39_, Mw ≈ 1526.5 g/mol, degree of deacetylation ≈ 85%, Sigma-Aldrich, St. Louis, MO, USA) and acetic acid (CH_3_COOH, 96% purity, Sigma-Aldrich, St. Louis, MO, USA) were used for polymer solution preparation. Streptomycin sulfate (STRS, C_21_H_4_1N_7_O_12_·H_2_SO_4_, Mw ≈ 728.7 g/mol, Panacea Biotec Ltd., Lalru, Punjab, India) was used as the antibacterial agent. All additional reagents used for the preparation of simulated body fluid (SBF) were of analytical grade and obtained from Sigma-Aldrich (St. Louis, MO, USA).

All chemicals and reagents were used as received without further purification unless otherwise specified.

### 2.2. Synthesis of (60s-Bg)

The bioactive glass with chemical composition (60% SiO_2_, 35% CaO, and 5% P_2_O_5_) was synthesized via the sol-gel method. 22.523 mL of tetraethyl orthosilicate (TEOS), 3.762 mL of 2 M nitric acid (HNO_3_), and 22.574 mL of deionized water were mixed. Thereafter, the mixture was allowed to hydrolyze for 30 min under acidic conditions to promote precursor stabilization prior to the sequential addition of the remaining components. The following chemicals were added one at a time, with 45 min between each one: 1.209 mL of triethyl phosphate (TEP) and 22.523 g of calcium nitrate tetrahydrate. The mixture was continuously stirred for 1.5 h until a homogeneous solution was obtained. The solution was dried at 120 °C for three days, evaporating all the water as steam. Subsequently, the powder was heated to 600 °C for three hours to eliminate the toxic nitrate ions and incorporate calcium into the silicate network [13,14]. An agate mortar was used to break up the BG grains. Then, a 90 µm filter was used to sort the BG granules [15,16].

### 2.3. Synthesis of Composite Scaffolds

**Figure 1:**
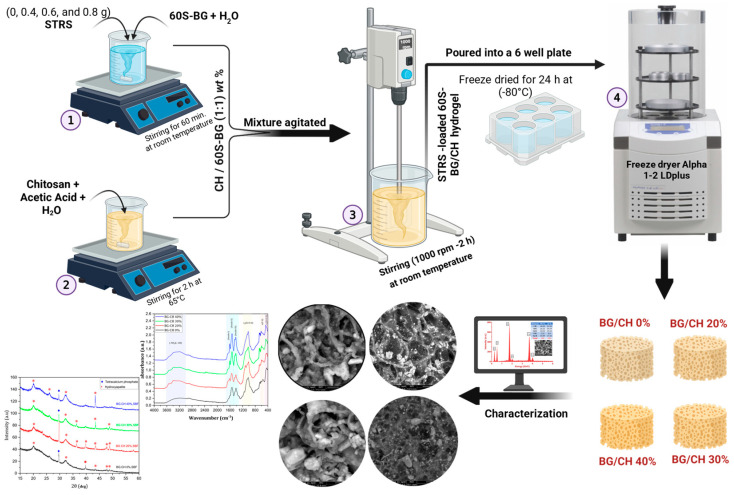
Flow chart of the synthesis of STRS-loaded 60S-BG/CH composite scaffolds, created in BioRender. Alesnawy, A. (2026). https://BioRender.com/fllddrl.

STRS-loaded 60S-BG/chitosan composite hydrogels were fabricated using the freeze-drying (lyophilization) technique (Figure 1). A constant weight ratio of 1:1 between bioactive glass and chitosan was maintained, while their absolute amounts were proportionally adjusted to accommodate increasing STRS loading (20–40 wt%), ensuring a constant total scaffold mass, as detailed in Table 1.

STRS-loaded scaffolds were prepared as the following steps: Different amounts of chitosan polymer (1, 0.8, 0.7, and 0.6 g) were dissolved in an aqueous 1% (*v*/*v*) acetic acid solution under continuous magnetic stirring at 65 °C for 2 h. Following this, different concentrations of STRS (0%, 20%, 30%, and 40% by weight) were incorporated into the 60S-BG solution and agitated at room temperature for a duration of one hour to facilitate initial dispersion. The STRS-60S-BG/CH precursor solutions were subsequently homogenized using a high-torque digital overhead stirrer (HT-50DX, Daihan Scientific Co., Wonju-si, Republic of Korea) at 969 rpm for 2 h to ensure complete mixing and uniform distribution of all components.

The homogeneous mixtures were placed into 6-well plates and underwent vacuum freeze-drying at −80 °C for a duration of 24 h. The freeze-drying process, recognized as a conventional method for scaffold fabrication, utilizes sublimation to eliminate water directly from the frozen state, thus maintaining the internal architecture of the material. The precursor hydrogels were frozen at −80 °C prior to lyophilization. Liquid nitrogen was used only during preliminary optimization trials to accelerate the freezing process; however, all scaffolds reported in the present study were fabricated under identical freezing conditions to ensure experimental consistency.

### 2.4. Study of Bioactivity In Vitro

The bioactive properties of 60S-BG/CH and STRS-loaded composite scaffolds were assessed in vitro by immersion in simulated body fluid (SBF), adhering to the established protocol by [17]. The samples were kept in sealed containers with SBF solution at a concentration of 2.5 g/L for 28 days at 37 °C. After being in the SBF for a while, the samples were taken out, rinsed with distilled water, and left to dry.

### 2.5. Antibacterial Activity

The antibacterial activity of the samples was evaluated by the agar diffusion method. The pathogenic microorganisms were obtained from the Faculty of Science (Boys) at Al-Azhar University (Cairo, Egypt). *Staphylococcus aureus* and *Enterococcus faecalis* are examples of Gram-positive bacteria, while *Escherichia coli* and *Klebsiella pneumoniae* are examples of Gram-negative bacteria [4] Samples (S0, S1, S2, and S3) were placed on nutrient agar plates that had been previously inoculated with the bacterial strains being tested: *Staphylococcus aureus*, *Enterococcus faecalis*, *Klebsiella pneumoniae*, and *Escherichia coli*. The plates were stored at 4 °C for 1 h to facilitate the diffusion of the tested materials into the agar medium before bacterial growth occurred. Following this, the plates were incubated at 37 °C for 24 h. All scaffold specimens used in the antibacterial assay were prepared with identical mass (rather than identical geometrical dimensions) to ensure consistent testing conditions during agar diffusion analysis.

Antibacterial activity was assessed by measuring the diameter of the inhibition zones (in millimeters) surrounding each sample. The appearance of a clear inhibition zone around the samples is an indication of the antibacterial activity of the BG/CH 20%, BG/CH 30%, and BG/CH 40% composite scaffolds [16,17].

### 2.6. In Vitro Release Study of (STRS)

The in vitro release behavior of streptomycin sulfate from composite scaffolds was investigated under non-sink conditions to evaluate the release characteristics and elucidate the underlying transport mechanism. Non-sink conditions were intentionally used to better simulate localized environments with limited fluid exchange. A constant mass of each STRS-loaded scaffold (0.25 g) was immersed in 100 mL of release medium, kept at 37 ± 0.5 °C under gentle orbital shaking (50 rpm) to ensure consistent diffusion conditions throughout the experiment.

Samples of 10 mL were taken from the release medium every 1 to 512 h (about 21 days) for testing. Sampling intervals were selected to capture both the initial burst release phase and the subsequent sustained release stage over the extended-release period.

Non-sink conditions were maintained throughout the experiment, as no fresh release medium was introduced to compensate for the withdrawn aliquots. We used a calibration curve that had already been made to measure the concentration of STRS in each aliquot using a spectrophotometer. The results were given in mg/mL [18,19]. Experiments were conducted in triplicate (*n* = 3), and results are presented as mean ± standard deviation. Statistical significance was evaluated using one-way ANOVA followed by Tukey’s post hoc test (*p* < 0.05).

The amount of STRS released at each sampling time ($M_{t}$) was calculated according to Equation (1).

(1) $$M_{t}=C_{t}\times V$$

where $C_{t}$ represents the STRS concentration (mg/mL) measured at time t, and V denotes the withdrawn volume (10 mL). The cumulative released amount was calculated by summing the released doses over successive time points. The cumulative release percentage was determined using Equation (2).

(2) $$\mathrm{Cumulative}\;\mathrm{release}\;(\%)=\frac{\sum M_{t}}{M_{0}}\times 100$$

where $M_{0}$ is the initial amount of STRS loaded into the scaffold.

### 2.7. Release Kinetics Analysis

To elucidate the release mechanism, the experimental release data were examined through various kinetic models, including zero-order, Higuchi, and Hixson–Crowell models. The zero-order model is expressed by Equation (3) [20].

(3) $$M_{t}=k_{0}t$$

The Higuchi model, which describes diffusion-controlled release from a matrix system, is given by Equation (4) [21].

(4) $$M_{t}\;=\;k_{H}t^{1/2}$$

The Hixson–Crowell model, which accounts for changes in surface area and matrix geometry during release, is described by Equation (5) [22].

(5) $$M_{0}^{1/3}-M_{t}^{1/3}=k_{HC}t$$

For early-stage release data (Mt/M∞ < 0.6), the Korsmeyer–Peppas model was applied using Equation (6) [23]. Kinetic model fitting was performed using linear regression analysis of the transformed model equations, and the goodness of fit was evaluated based on the correlation coefficient (R^2^).

(6) $$\frac{M_{t}}{M_{\infty }}=kt^{n}$$

where k is the kinetic constant and n is the diffusion exponent indicative of the release mechanism. The half-release time ($t_{50}$) was calculated from the fitted kinetic parameters. Statistical analysis was performed using IBM SPSS Statistics version 27 (IBM Corp., Armonk, NY, USA), and differences were considered statistically significant at *p* < 0.05.

### 2.8. Characterization

STRS-loaded 60S-BG/CH composite scaffolds were characterized before and after immersion in simulated body fluid (SBF) using several physicochemical techniques. Fourier transform infrared spectroscopy (FTIR, Nicolet 6700, Thermo Scientific, Waltham, MA, USA) was used to identify the functional groups. Spectra were recorded in the range of 4000–400 cm^−1^ with a resolution of 4 cm^−1^ using the KBr pellet method (1 mg sample/200 mg spectroscopy-grade KBr, Merck, Germany). The crystalline structure was analyzed by X-ray diffraction (XRD, D8 ADVANCE, Bruker, Germany) using Cu Kα radiation (λ = 1.54060 Å) and a nickel filter. Patterns were collected over a 2θ range of 0–60° with a step size of 0.014° and a counting time of 1 s per step. Surface morphology was examined using scanning electron microscopy (SEM, XL30, Philips) operated at an accelerating voltage up to 30 kV. Elemental composition was determined by energy-dispersive X-ray analysis (EDXA) coupled with SEM using a 30 mm^2^ Si(Li) R-RSUTW detector at 15 kV. EDXA spectra were collected from representative surface regions of the scaffold samples selected during SEM examination to evaluate the elemental composition of the investigated composite structures. Streptomycin release was quantified using UV-Vis spectroscopy (JASCO V-630) brought from JASCO Corporation (Tokyo, Japan).

## 3. Results

### 3.1. XRD Before and After SBF

**Figure 2:**
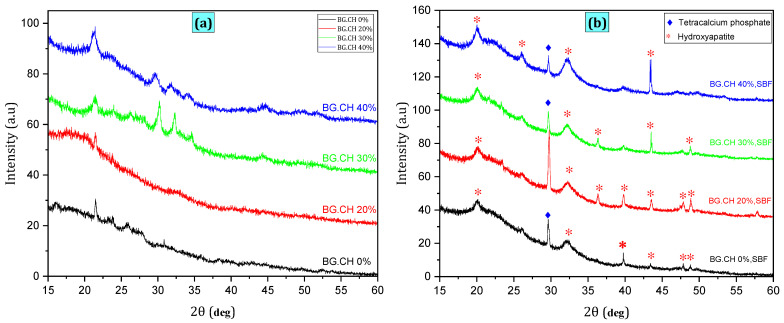
(XRD) patterns of STRS-loaded BG/CH biocomposite scaffolds (**a**) before and (**b**) after immersion in SBF for 28 days.

Figure 2a shows the results of the XRD analysis of BG/CH biocomposite scaffolds and STRS-loaded CH/BG scaffolds (20%, 30%, and 40%) before they were soaked in SBF. The presence of the main characteristic peak (2θ = 21.5°) of chitosan indicates that it was in a crystalline state [24,25]. The characteristic chitosan diffraction peak gradually decreased in intensity with increasing STRS loading, suggesting increased interactions between STRS molecules and the BG/CH matrix.

Figure 2b presents the XRD patterns of the CH/BG biocomposite scaffolds after they were immersed in simulated body fluid (SBF) for 28 days. The diffraction patterns indicate that crystalline hydroxyapatite (HAp) has formed on the surfaces of the scaffolds. The (200) and (112) planes of HAp (JCPDS #09-0432) are observed in two distinct diffraction peaks at around 21° and 32° [26]. Additional weak peaks appearing at about 2θ ≈ 39°, 43°, and 48° are related to the (310), (113), and (320) planes of HAp. The HAp diffraction peaks increase in intensity as the STRS concentration goes up, indicating that more apatite is forming on the scaffold surfaces. There is also a diffraction peak at 2θ ≈ 29.6° in all the samples. This peak is attributed to tricalcium phosphate (TeCP) [27,28]. The peak intensity slowly goes down as the STRS content goes up, while the HAp peaks increase in intensity. This trend indicates that TeCP is being gradually consumed while apatite is simultaneously forming during immersion in SBF.

### 3.2. FTIR Before and After SBF

FTIR spectroscopy was used to evaluate the interactions between the polymer (CH), the particles (60S-BG), and STRS, as well as the formation of an HAp layer on the surface of STRS-loaded CH/BG biocomposite scaffolds (BG-CH 20%, BG-CH 30%, and BG-CH 40%) after immersion in SBF.

**Figure 3:**
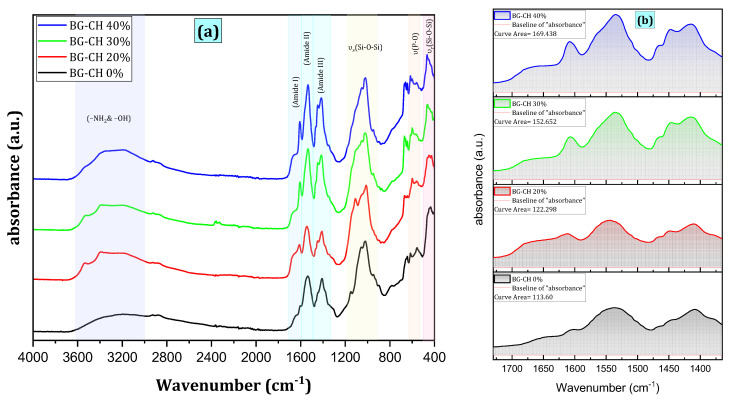
(**a**): FTIR patterns of STRS-loaded BG/CH biocomposite scaffolds before immersion in SBF, (**b**): Area under the curve in the range 1365–1730 cm^−1^.

FTIR spectra before immersion in SBF: the characteristic bands of both CH/BG (BG-CH 0%) and STRS-loaded CH/BG (BG-CH 20%, BG-CH 30%, and BG-CH 40%) biocomposite scaffolds are observed in Figure 3. However, several characteristic bands are shifted, deformed, or disappear. This behavior is attributed to chemical interactions between the bioactive glass, chitosan, and streptomycin sulfate. Both samples mainly exhibit the stretching and bending vibrations of the Si–O–Si bridge.

The bending vibrations of the Si–O–Si and O–Si–O links are assigned to characteristic bands between 400 and 500 cm^−1^ [29,30,31]. After the addition of STRS, the rocking vibration of Si–O–Si (r) shifts to a higher wavenumber, from 458.9 cm^−1^ to 465 cm^−1^. A weak band in the range of 760–810 cm^−1^ corresponds to the stretching vibrations of the O–Si–O bonds, while the increased intensity of bands in the range of 1000–1100 cm^−1^ is attributed to the symmetric stretching vibration of the Si–O–Si bonds in BG [32] In addition, weak bands at 608 cm^−1^ and 667 cm^−1^ are attributed to the asymmetric and stretching vibrations, respectively, of P–O–P [30,31]. Additionally, a shoulder at 946 cm^−1^ is attributed to the non-bridging Si–O–X (X = Ca, H) bonds in BG [14].

The weak band located at ~1380 cm^−1^ can be attributed to CH_3_ in the amide group [33]. The shoulder peak for the asymmetric stretching of C–O–C is found at around 1150 cm^−1^ in chitosan. Three characteristic bands of chitosan at 1650, 1558, and 1314 cm^−1^ are attributed to C=O–NH–CH_2_, N–H, and C–N (Amide I, Amide II, and Amide III), respectively [26]. The adsorption of STRS onto chitosan and BG did not show any significant additional peaks because similar functional groups cause their peaks to overlap in the FTIR spectra of raw BG and chitosan.

The bands at 2926 and 2880 cm^−1^ correspond to the -CH- bending vibrations. The broad absorption band at 3000–4000 cm^−1^ is attributed to the stretching vibrations of –NH_2_ and –OH groups [34,35]. STRS is well incorporated into the matrix of 60S-BG/CH to form the scaffolds of STRS-loaded 60S-BG/CH composite. As can be seen, the peak at 3430 cm^−1^ appears due to the stretching vibration of –OH groups (BG-CH 0%). After the addition of STRS, the vibration shifts to a lower wavenumber, with a broader peak at 3423, 3422, 3421, and 3420 cm^−1^ for BG-CH 20%, BG-CH 30%, and BG-CH 40%, respectively. These changes in band intensity and position confirm the successful incorporation of the STRS component into these CH/BG scaffolds.

**Figure 4:**
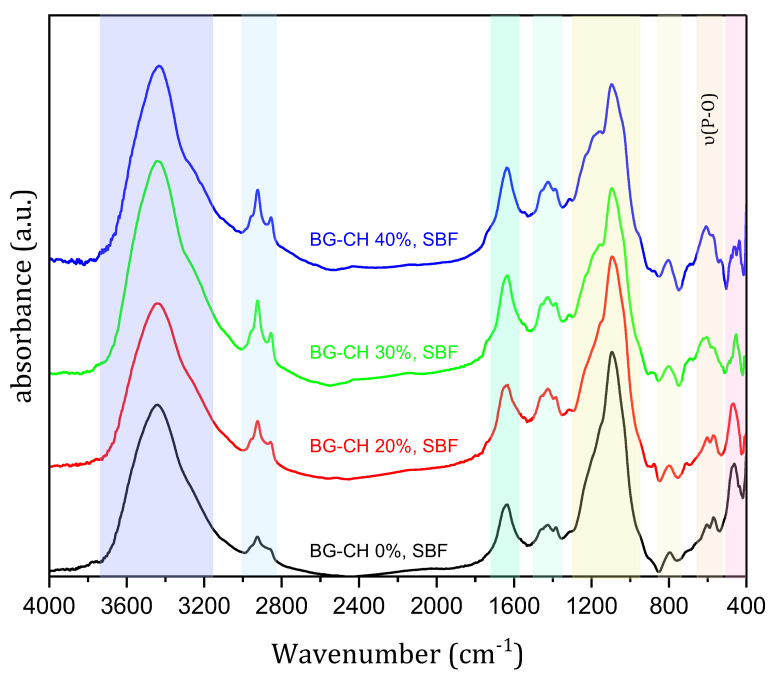
(FTIR) patterns of STRS-loaded BG/CH bio-composite scaffolds after immersion in SBF for 28 days.

FTIR spectra after immersion in SBF (Figure 4) show that new peaks at 606 and 569 cm^−1^ (*δ* P–O for crystal) are observed, further supporting the formation and growth of HAp [14]. It is generally accepted that the bioactivity of a material can be evaluated by HAp formation [15]. While SBF immersion is widely used as a preliminary method to evaluate in vitro bioactivity through hydroxyapatite formation, it does not fully represent the biological response under physiological conditions. Therefore, the STRS-loaded CH/BG composite scaffolds exhibit in vitro bioactivity. These increased and newly appeared phosphate-related bands further confirm the precipitation and growth of hydroxyapatite (HAp) on the scaffold surfaces after immersion in SBF. However, further cytocompatibility and cellular studies remain necessary to confirm the biological suitability of the developed scaffolds for bone tissue engineering applications.

### 3.3. SEM of the Composite Scaffolds Before and After SBF

**Figure 5:**
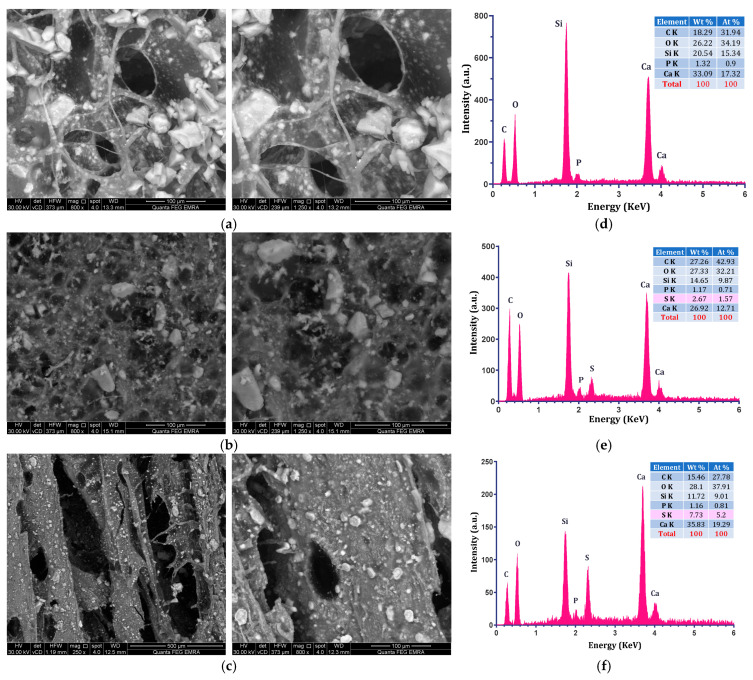
(SEM) images of, (**a**) BG-CH 0%, (**b**) BG-CH 20%, (**c**) BG-CH 40%, and (EDXA) profiles of (**d**) BG-CH 0%, (**e**) BG-CH 20%, (**f**) BG-CH 40%. Before immersion in SBF. Different magnifications were intentionally used to highlight distinct morphological features and pore characteristics observed within each scaffold composition.

Figure 5 shows the scanning electron microscopy (SEM) images and energy dispersive X-ray analysis (EDXA) results of BG/CH biocomposite scaffolds and STRS-loaded BG/CH scaffolds (20% and 40%). Representative SEM micrographs were selected to illustrate the main morphological variations observed between unloaded and STRS-loaded scaffold formulations.

The BG/CH biocomposite scaffold displayed a microporous membrane structure with interconnected open pores, as shown in Figure 5a. Although SEM analysis confirms the presence of interconnected porous structures, quantitative characterization of porosity, including pore size distribution and total porosity percentage, will be addressed in future work using advanced techniques such as micro-computed tomography (micro-CT) or mercury intrusion porosimetry. SEM also revealed the presence of a smooth distribution of bioglass particles in various areas on the surface. The morphology of STRS-loaded BG/CH scaffolds (20%) showed a porous membrane structure, but the size of this micropore partially decreased with increasing STRS content, as shown in Figure 5b, and further decreased in the STRS-loaded CH/BG scaffolds (40%) sample. The appearance of larger cavities in the higher STRS-loaded scaffold may be attributed to compositional changes induced by increased STRS incorporation, which could alter matrix density and influence ice crystal formation during the freeze-drying process. Consequently, higher STRS loading may contribute to the formation of larger pore domains and more heterogeneous porous morphology. The reduction in pore size with increasing STRS content indicates increased matrix density, which directly influences diffusion pathways and contributes to sustained drug release behavior.

In EDXA elemental analysis of the STRS-loaded BG/CH scaffolds (20% and 40%), the intensity of the peak assigned to sulfur (S) was higher for BG-CH 40% than BG-CH 20%, thus confirming the higher degree of STRS substitution in the prepared BG/CH biocomposite scaffolds as intended (see Figure 5e,f).

**Figure 6:**
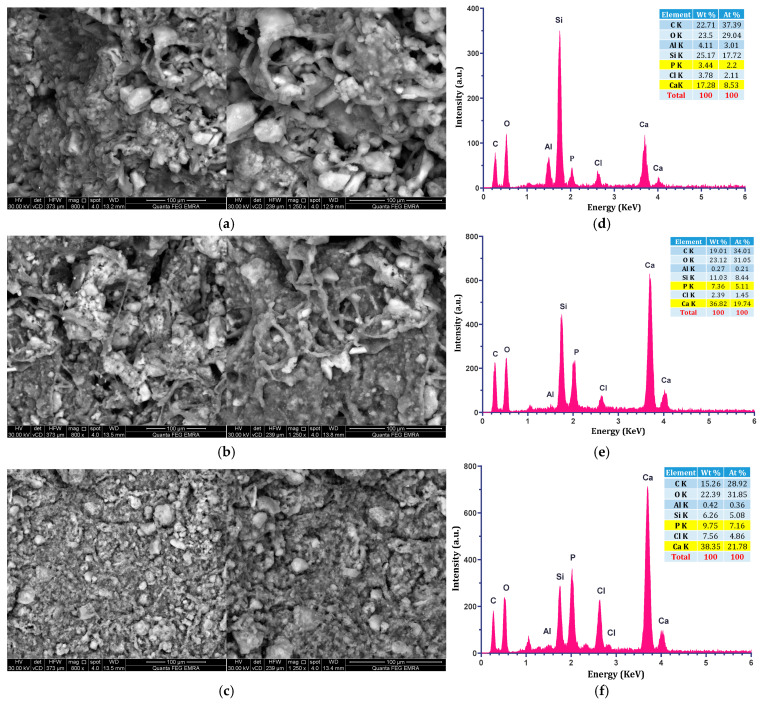
Scanning Electron Microscopy (SEM) images of, (**a**) BG-CH 0%, (**b**) BG-CH 20%, (**c**) BG-CH 40%, and EDXA profiles of (**d**) BG-CH 0%, (**e**) BG-CH 20%, (**f**) BG-CH 40% After immersion in SBF.

Figure 6 shows SEM images and EDXA profiles of BG-CH 0%, BG-CH 20%, and BG-CH 40% biocomposite scaffolds after immersion in SBF for 28 days. Results showed that a layer of flake-like crystals of HAp was deposited on the scaffold surface. Briefly, HAp partially covered the surface of the (BG-CH 0%) sample, but the thickness of this layer significantly increased on the (BG-CH 20%) sample and almost completely covered the surface of (BG-CH 40%). Although elemental mapping analysis was not performed in the present study, the EDXA spectra confirmed the presence and relative increase of sulfur-containing STRS within the composite scaffolds. More detailed spatial elemental distribution analysis will be considered in future investigations.

EDXA elemental intensities from Figure 6a–c were converted into wt% and are presented in Figure 6d–f. After immersion in SBF for 28 days, EDXA results showed an increase in Ca and P concentrations compared with the silicon content. The Si content on the surface of the scaffold samples became extremely low due to the formation of the HAp layer. Ca and P concentrations increased in the BG-CH 40% sample (38.35 and 9.75 wt%) compared with their concentrations in BG-CH 20% (36.82 and 7.36 wt%) and BG-CH 0% (17.28 and 3.44 wt%), respectively. It was observed that a sample containing a high amount of STRS (40%) exhibited a thicker HAp layer due to its high porosity. This behavior may be indirectly associated with changes in scaffold morphology and surface characteristics induced by STRS incorporation, which could facilitate ion exchange and apatite nucleation during SBF immersion.

### 3.4. Bacterial Sensitivity of Composite Scaffolds

**Figure 7:**
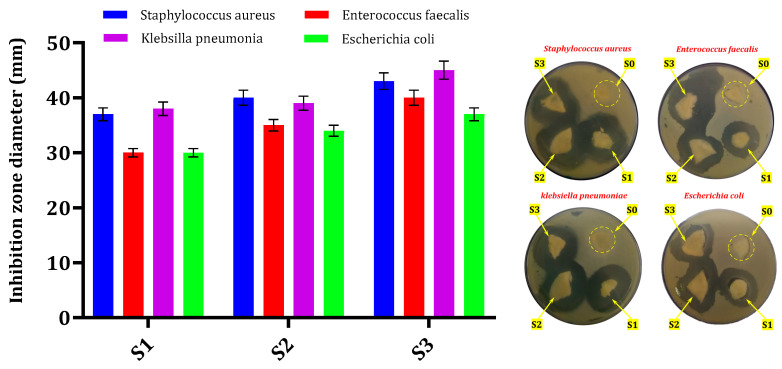
Antibacterial activity of STRS-loaded BG/CH composite scaffolds using the agar diffusion method.

Figure 7 shows a clear inhibition zone resulting from the release of streptomycin sulfate from STRS-loaded BG/CH composite scaffolds. The agar diffusion method was used to evaluate the antibacterial activity of these scaffolds against Gram-positive bacteria (*Staphylococcus aureus* and *Enterococcus faecalis*) and Gram-negative bacteria (*Escherichia coli* and *Klebsiella pneumoniae*). The presence of a distinct inhibition zone surrounding the sample demonstrates the effective performance of the STRS-loaded BG/CH composite scaffolds as a drug delivery system, facilitating the sustained release of the antibiotic streptomycin sulfate. This release mechanism is specifically intended to target pathogenic microorganisms, consequently enhancing the antibacterial activity against these organisms [36].

The unloaded scaffold (S0) showed no inhibitory zones, which means that the antibacterial action is mostly due to the presence of streptomycin sulfate. The drug-free scaffold (S0) was used as a control, confirming that antibacterial activity is primarily associated with STRS incorporation. The diameters of the zone around samples (S0, S1, S2, and S3) are (0, 37, 40, and 43 mm) for *Staphylococcus aureus*, (0, 30, 35, and 40 mm) for *Enterococcus faecalis*, (0, 30, 34, and 37 mm) for *Escherichia coli*, and (0, 38, 39, and 45 mm) for *Klebsiella pneumoniae*, as illustrated in Table 2. The inhibition zone diameter increased progressively with increasing STRS loading, indicating a concentration-dependent enhancement in antibacterial activity. Among all investigated formulations, the S3 scaffold exhibited the highest antibacterial efficacy, with inhibition zone diameters reaching up to 45 mm. The observed differences in antibacterial susceptibility between Gram-positive and Gram-negative bacteria may be attributed to variations in their cell wall structure and membrane permeability. The increase in inhibition zone diameter with increasing STRS loading correlates strongly with the sustained release profile, indicating prolonged antibacterial activity.

The results reveal that the composite scaffolds have substantial localized antibacterial activity. These findings demonstrate promising antibacterial properties and sustained-release characteristics in the developed scaffold system.

### 3.5. Uv–Vis Spectroscopy

#### 3.5.1. Determination of the Characteristic Absorption Peaks

**Figure 8:**
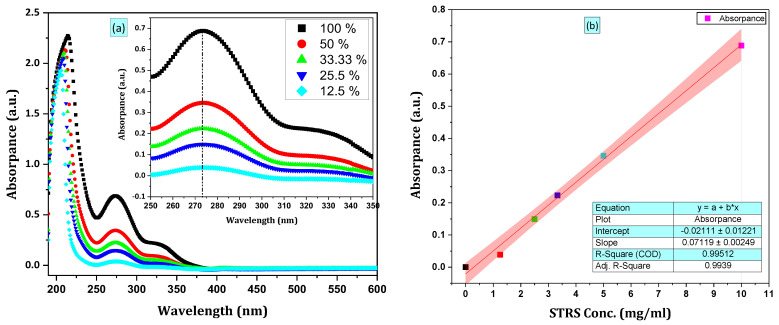
(**a**) UV-visible absorption spectra of STRS concentration, (**b**) Calibration curve of the released drug STRS.

Figure 8a illustrates the UV–visible absorption spectra for a solution of Streptomycin Sulfate (STRS). The absorption peak intensities of STRS were observed at 273, 274, 274, 274, and 273 nm, in that order. The main absorption peak for the STRS solution was observed at a wavelength of λmax = 274 nm. This wavelength was subsequently selected for quantitative UV–Vis analysis and calibration curve construction.

##### Calibration Curve of the Released Drug STRS

Multiple standard drug solutions at the maximum absorption wavelength (λmax = 274 nm), corresponding to the STRS drug, were used to calibrate the UV–Vis absorption spectroscopy (JASCO v-630) instrument. Aliquots (100, 50, 33.3, 25.5, and 12.5%) of the stock solution were diluted with 2 mL of distilled water at pH 7.4 to prepare concentrations ranging from 12.5 to 100 mg/mL. The absorbance of these solutions was determined at 274 nm using a UV–Vis spectrophotometer, as shown in Table 3. The calibration curve of the drug was fitted to a straight line with a correlation coefficient (R^2^) = 0.99512 for STRS, as shown in Figure 8b.

#### 3.5.2. Determination of the Amount of Drug Released

In vitro release experiments were conducted to evaluate the release behavior of streptomycin sulfate (STRS) from BG/CH biocomposite scaffolds containing 20%, 30%, and 40% STRS over a period extending to 512 h. Table 4 demonstrates a progressive increase in cumulative drug release for all investigated formulations throughout the experimental period. An initial burst release was observed during the early release intervals, which may be attributed to the diffusion of STRS molecules located near the scaffold surface. This phase was followed by a sustained release stage characterized by gradual diffusion of the incorporated drug from the internal scaffold matrix into the surrounding medium.

### 3.6. In Vitro Release Behavior of STRS

**Figure 9:**
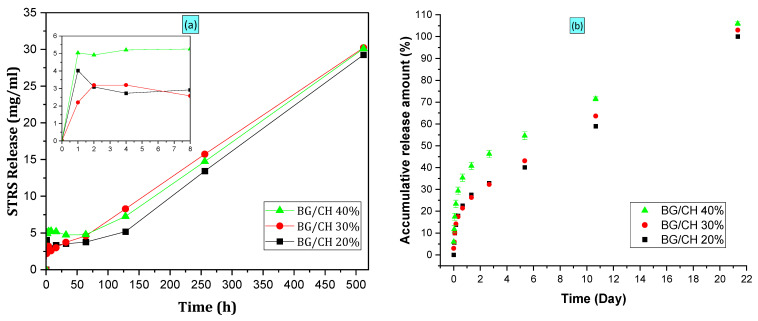
(**a**) The amount of released STRS concentration from samples at 512 h, (**b**) release profile of STRS in terms of the percentage (%) of STRS release as a function of time.

Figure 9a,b show the release profiles in both the total amount and the total percentage. The release profile follows a biphasic pattern consisting of an initial burst release followed by sustained diffusion-controlled release. Such biphasic release behavior is commonly observed in porous polymeric delivery systems, where surface-associated drug molecules contribute to the initial burst release, followed by sustained diffusion from the internal scaffold matrix [37].

The BG-CH 40% scaffold consistently exhibited higher cumulative release values compared with BG-CH 20% and BG-CH 30%, particularly at intermediate release intervals. This behavior may be attributed to the increased concentration gradient associated with higher STRS loading, which enhances diffusion-driven transport within the scaffold matrix [38].

### 3.7. Cumulative Release and Statistical Evaluation

Figure 9b shows the cumulative release profiles demonstrated a time-dependent increase in STRS release, ultimately reaching complete release for all formulations by the end of the experimental period. There were statistically significant differences in cumulative release between the drug loadings (*p* < 0.05) (Table 4). This conclusion indicates that the scaffold’s composition significantly affects drug release. As the STRS levels increased (20%, 30%, and 40%), the half-time of release (t_50_) values decreased (195.2, 189.7, and 138.7 h, respectively). The observed trend corresponds with the notion that systems exhibiting elevated initial drug concentrations serve as a more potent driving force for diffusion [38].

### 3.8. Release Kinetics and Mechanistic Interpretation

We examined the cumulative release data to elucidate the fundamental mechanisms of release by employing various empirical and semi-empirical kinetic models, including zero-order, Higuchi, Hixson–Crowell, and Korsmeyer–Peppas models.

Table 5 provides a summary of the kinetic parameters and correlation coefficients. The zero-order model, which posits a constant release rate independent of concentration, did not fit well (R^2^ ≈ 0.88–0.96) and failed to account for the initial burst behavior. Consequently, pure zero-order kinetics does not fully characterize the system [39].

The Higuchi model provided the best fit (R^2^ ≈ 0.97–0.98), confirming diffusion-controlled release. Higuchi’s classic formulation states that the total drug release is directly proportional to the square root of time. This indicates that the transport mechanism primarily relies on diffusion, regulated by concentration gradients within the matrix [38].

The Hixson–Crowell model, which studies how the shape of the matrix and surface area change during release, had lower R^2^ values than Higuchi. These findings suggest that matrix erosion and geometrical changes contributed minimally to the overall release behavior of the developed scaffolds.

The Korsmeyer–Peppas model provided additional insight into the release mechanism by correlating fractional drug release with a power-law expression capable of distinguishing different transport phenomena within polymeric systems. The calculated n-values (<0.5) indicate Fickian diffusion as the dominant release mechanism. The calculated release exponent (n) values ranged from approximately 0.378 to 0.482 across the formulations, indicating Fickian diffusion within a polymeric matrix framework [39]. These findings indicate that drug molecules primarily diffuse through the hydrated BG/CH matrix, with minimal impact from polymer relaxation or erosion processes.

The kinetic analysis shows that Fickian diffusion is the main way that STRS is released from BG/CH scaffolds. This is consistent with models frequently utilized in the controlled release literature [40]. The observed variations in kinetic constants and diffusion exponent values demonstrate the tunable release characteristics of the developed scaffold system through modulation of STRS loading content.

## 4. Conclusions

Streptomycin sulfate (20–40%) was effectively integrated into bioactive glass/chitosan (BG/CH) composite scaffolds produced through an optimized unidirectional freeze-drying method, yielding a structurally continuous matrix with a highly interconnected porous architecture.

Comprehensive structural, chemical, and morphological assessments utilizing X-ray diffraction (XRD), Fourier-transform infrared spectroscopy (FTIR), scanning electron microscopy (SEM), and energy-dispersive X-ray analysis (EDXA) validated the stability, uniform composition, and retention of physicochemical stability and apatite-forming behavior of the scaffolds prior to and following in vitro evaluation.

The STRS–BG/CH scaffolds demonstrated sustained antibacterial activity against both Gram-positive and Gram-negative bacteria, as well as improved apatite formation in vitro. Drug release was predominantly governed by diffusion-controlled mechanisms, as confirmed by kinetic modeling. The STRS–BG/CH scaffolds demonstrated sustained antibacterial activity against both Gram-positive and Gram-negative bacteria, together with enhanced apatite-forming behavior in vitro. Drug release was predominantly governed by diffusion-controlled mechanisms, as confirmed by kinetic modeling. The findings indicate that scaffold architecture and composition significantly influence antibacterial performance, release behavior, and apatite-forming characteristics. Overall, the present work provides a methodological and physicochemical investigation of STRS-loaded BG/CH scaffold systems with promising antibacterial and release properties. Nevertheless, further biological investigations, including cytocompatibility and cellular response studies, remain necessary before confirming suitability for bone tissue engineering applications.

## Figures and Tables

**Table 1:** Chemical composition of Two grams of STRS-loaded BG /CH Scaffolds in (wt%).

Sample Code	Sample Name	60S-BG/CH (1:1) wt%	CH (g)	60S-BG (g)	STRS	
					wt%	(g)
S0	CH-BG 0%	100%	1	1	0%	0
S1	CH-BG 20%	80%	0.8	0.8	20%	0.4
S2	CH-BG 30%	70%	0.7	0.7	30%	0.6
S3	CH-BG 40%	60%	0.6	0.6	40%	0.8

**Table 2:** Microbial sensitivity of STRS-loaded CH/BG composite Scaffolds against pathogenic tested microorganisms.

Sample Code	Mean Inhibition Zone Diameter (mm) Against Test Pathogens			
	*Staphylococcus aureus*	*Enterococcus faecalis*	*Escherichia coli*	*Klebsiella pneumoniae*
S0	0	0	0	0
S1	37 ± 1.041	30 ± 0.601	30 ± 0.882	38 ± 0.601
S2	40 ± 0.416	35 ± 0.727	34 ± 0.504	39 ± 0.866
S3	43 ± 0.441	40 ± 1.014	37 ± 1.155	45 ± 1.044

**Table 3:** Standard absorption values of STRS in distilled water, pH 7.4, with different concentrations.

No	Concentration (mg/mL)	Absorbance (%)
1	10	0.68835
2	5	0.34606
3	3.33	0.22311
4	2.5	0.14927
5	1.25	0.03837

**Table 4:** The Percentage of Streptomycin sulfate (STRS) drug released from samples.

Time (h)	BG-CH 20%		BG-CH 30%		BG-CH 40%	
	mg/mL	%	mg/mL	%	mg/mL	%
0	0	0	0	0	0	0
1	4.030	5.65	2.201	2.869	5.036	5.776
2	3.082	9.97	3.188	7.023	4.916	11.416
4	2.727	13.81	3.194	11.185	5.205	17.386
8	2.916	17.89	2.575	14.540	5.255	23.414
16	3.339	22.58	2.990	18.437	5.177	29.353
32	3.546	27.55	3.744	23.316	4.735	34.783
64	3.760	32.83	4.606	29.319	4.777	40.263
128	5.189	40.11	8.286	40.117	7.260	48.590
256	13.439	58.96	15.730	60.616	14.728	65.483
512	29.253	100	30.221	100	30.093	100
Total amount	71.28		76.74		87.18	
*p* value *	0.001		0.001		0.002	

* *p*-value < 0.05 was considered statistically significant. (Statistical software IBM—SPSS, version 27.0).

**Table 5:** Release kinetics parameters of different STRS-loaded BG/CH biocomposite scaffold.

Formula Code	Zero-Order		Higuchi Release Model		Hixson-Crowell Release Model		Korsmeyer-Peppas Model			
	R^2^-Value	*k*_0_ (mg·h^−1^)	R^2^-Value	*k_H_* (mg·h^−½^)	R^2^-Value	*k_HC_* (mg^1/3^·h^−1^)	R^2^-Value	*n*	k (h^−n^)	*t*_50_ (hours)
BG-CH 20%	0.9421	0.1243	0.9705	2.7746	0.9242	0.0071	0.9715	0.3780	0.0723	195.1576
BG-CH 30%	0.9568	0.1398	0.9808	3.1536	0.9358	0.0074	0.9313	0.4818	0.0441	189.7115
BG-CH 40%	0.8828	0.1495	0.9765	3.3937	0.9386	0.0076	0.9236	0.4039	0.0823	138.6837

*k*_0_: zero-order release constant; *k_H_*: Higuchi diffusion constant; *k_HC_*: Hixson–Crowell release constant; *n*: release exponent calculated from the Korsmeyer–Peppas model using the initial 60% of drug release; *k*: Korsmeyer–Peppas kinetic constant; *t*_50_: time required to release 50% of the total loaded drug.

## Data Availability

All data generated or analyzed during this study are included in this published article.
